# Spontaneous Repositioning of a Traumatic Intraorbital Encephalocele

**DOI:** 10.1097/SCS.0000000000011512

**Published:** 2025-05-23

**Authors:** Tanya Wolffenbuttel, Nadine Denneman, Sander Idema, Dyonne Hartong, Peerooz Saeed, Leander Dubois

**Affiliations:** * Department of Neurosurgery, Amsterdam University Medical Center; † Department of Ophthalmology, Amsterdam University Medical Center; ‡ Department of Oral and Maxillofacial Surgery, Amsterdam University Medical Center, Amsterdam, The Netherlands

**Keywords:** Neurotrauma, orbital roof fracture, superior orbital fissure syndrome, traumatic intraorbital encephalocele

## Abstract

Fractures of the orbital roof are relatively rare facial fractures that are often caused by high-energy trauma and are frequently seen in combination with intracranial injury and other facial fractures. Surgical reconstruction should be considered in cases with threatened visual function, significant displacement of fracture fragments, dural laceration, or additional facial fractures requiring repositioning. A rare complication of an orbital roof fracture, described in only a few case reports, is a traumatic intraorbital encephalocele (TIOE). This occurs when intracranial swelling disrupts the equilibrium between intracranial pressure (ICP) and intraorbital pressure, causing downward pressure and herniation of brain tissue through the fracture in the orbital roof. This can lead to compression and dysfunction of intraorbital structures, such as cranial nerves III, IV, and VI, which enter the orbit through the superior orbital fissure. The treatment of a TIOE requires a multidisciplinary approach involving neurosurgery, oral and maxillofacial surgery, and ophthalmology. In this article, the case of a 23-year-old male with extensive facial fractures after a motor vehicle collision is presented. Progressive displacement of the left orbital roof is observed as a TIOE develops in the early post-trauma phase. Remarkably, spontaneous repositioning of the orbital roof and reduction of the TIOE occurred over the following weeks under conservative management as intracranial swelling subsided, followed by remodeling.

A 23-year-old male is brought into the emergency room after being struck by a motor vehicle at high speed. Prehospital endotracheal intubation is performed because of a decreased level of consciousness. He has extensive external facial injuries, including periorbital ecchymosis. On ophthalmologic evaluation, pupillary light reflexes and intraocular pressure (IOP) are normal. In addition, the eye position is straight, there is no evident proptosis, and the eyelids, despite the hematoma in the upper and lower eyelid, are not overly tense and can easily be opened. Both eyes are intact with round pupils, normal anterior chamber anatomy and a clear view of the retina. A CT scan shows intracranial contusion, primarily in the left frontal lobe, and extensive facial fractures involving both orbits (Fig. 1). A conservative approach is taken, and the patient is admitted to the intensive care unit.

**FIGURE 1:**
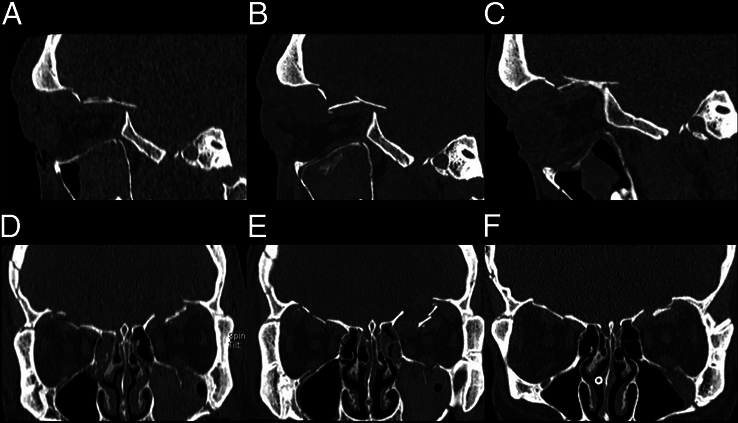
Computed tomography (CT) scans of the described case on day 0 (A, D), day 5 (B, E), and day 18 (C, F) demonstrating extensive facial fractures and evolution of left orbital roof position.

The patient regains consciousness over the following days, and periorbital swelling gradually subsides. However, the degree of exophthalmos seems to increase, and an abnormal lateral displacement of the left eye is noted (Fig. 2). This is accompanied by restriction of eye movement in all directions without ptosis or mydriasis. These findings could be due to increased intraorbital swelling causing mechanical limitation or palsy of one or more cranial nerves responsible for ocular motility (CN III, IV, and VI). A cranial nerve palsy may directly result from intracranial trauma or occur secondary to increased intracranial or intraorbital pressure. To differentiate between these etiologies, a new CT scan is performed, which shows no progression of intracranial injury but does reveal increased displacement of the left orbital roof (Fig. 1). There is no impingement of extraocular muscles (EOM), eyelid tension remains stable despite increased exophthalmos, and IOP remains within normal range. No urgent intervention is performed without signs indicating an acute risk to visual function. An additional MRI scan reveals bilateral intraorbital hematoma and herniation of brain tissue through the fracture in the left orbital roof (Fig. 3). This rare finding is referred to as a traumatic intraorbital encephalocele (TIOE). On the basis of the abnormal ophthalmologic findings (progressive exophthalmos with restricted eye movement) that seem to be caused by local compression from the TIOE, the decision is made to schedule a surgical reconstruction of the displaced orbital roof.

**FIGURE 2:**
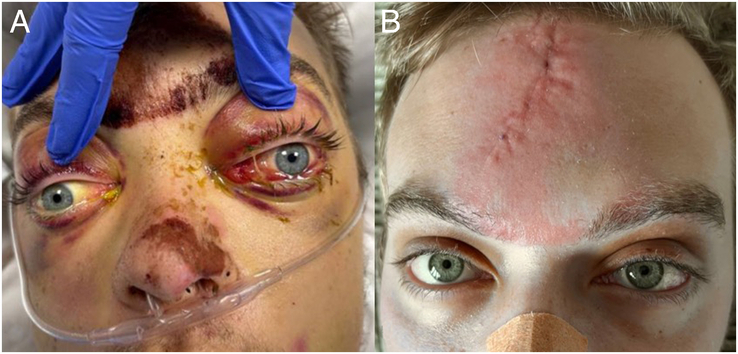
External facial injuries and ocular alignment. On day 5 (A) abnormal lateral displacement of the left eye is noted, which normalizes by day 25 (B).

**FIGURE 3:**
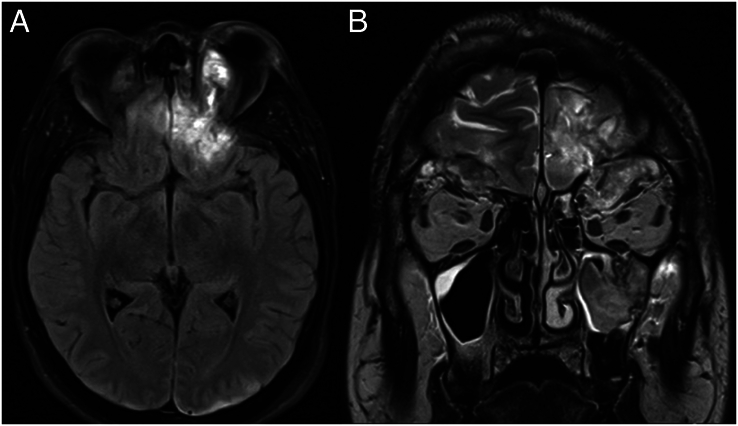
MRI scan on day 9 post-trauma demonstrating the traumatic intraorbital encephalocele in axial (A) and coronal (B) planes.

In preparation for surgery, a new CT scan is performed ~3 weeks post-trauma (Fig. 1). Remarkably, reduced swelling or atrophy in the left frontal lobe and decreased downward pressure have resulted in spontaneous repositioning of the left orbital roof to an almost anatomical position. On the basis of these findings, surgery is postponed, and watchful waiting is initiated. One week after the last CT scan, ocular alignment and motility return to normal (Fig. 2). The patient is subsequently discharged from the hospital. After 3 months, an orthoptic examination confirms that ocular motility has remained unaffected.

## DISCUSSION

The orbit functions as a protective shield for the ocular globe and other intraorbital structures, such as cranial nerves III, IV, and VI, and the extraocular muscles (EOM) that regulate ocular motility.^1^ The most common orbital fractures involve the orbital floor and/or medial wall. Fractures of the orbital roof are relatively rare, with an incidence of 1% to 9% of all facial fractures.^2^ These fractures are usually caused by high-energy trauma and are often seen in combination with other facial fractures and intracranial injuries. Isolated orbital roof fractures are relatively more common in children under 5 as pneumatization of the frontal sinus, which acts as a cushion for the orbital roof, has yet to occur. Clinical signs that can indicate an orbital roof fracture include periorbital hematoma and/or edema, subconjunctival ecchymosis, proptosis, hypoglobus, decreased visual acuity, restricted ocular motility, and ptosis.^3^

International consensus regarding the management of orbital roof fractures has yet to be reached. Generally, a conservative approach is justified for isolated fractures with good alignment and normal intraorbital pressure, without abnormalities on ophthalmologic examination or signs indicating dural laceration (rhinorrhea, oculorrhea).^3–5^ The extent and direction of orbital roof displacement depend on the intracranial and intraorbital pressure equilibrium. If intraorbital pressure increases due to edema or hemorrhage, the orbital roof shifts in a superior direction, resulting in a blow-out fracture. Conversely, intracranial swelling will lead to the displacement of the orbital roof towards the orbit, causing a blow-in fracture.^6^ The traditional approach towards blow-in fractures of the orbital roof was surgical reconstruction to prevent damage to intraorbital structures. In the past decade, multiple studies have advocated for a conservative approach as the orbital roof tends to spontaneously reposition once intracranial swelling subsides, and the equilibrium between ICP and intraorbital pressure is restored, followed by remodeling.^6,7^ Unlike the case presented in this article, the abovementioned studies focused exclusively on asymptomatic patients without neurosurgical complications such as a dural laceration or a TIOE.

In blow-in fractures of the orbital roof, displacement of bone fragments towards the superior orbital fissure can cause a localized pressure increase, a condition known as superior orbital fissure syndrome (SOFS). This rare but serious complication is characterized by partial or complete dysfunction of cranial nerves that enter the orbit through the superior orbital fissure (CN III, IV, V_1_, and VI). Clinical symptoms include ptosis, mydriasis, upper eyelid and forehead anesthesia, and restricted ocular motility.^8,9^ If SOPS is suspected and imaging confirms displacement of bone fragments towards the superior orbital fissure, surgical repositioning of fragments is recommended with intraoperative administration of corticosteroids to reduce reactive edema.^10^ Another complication associated with traumatic orbital injury is orbital compartment syndrome (OCS), an ophthalmologic emergency that can promptly result in irreversible vision loss. In OCS, rapidly progressive intraorbital hemorrhage or another type of expanding lesion can lead to an acute rise in intraorbital pressure. The increased intraorbital pressure is initially compensated by an anterior globe displacement, also known as exophthalmos. If this mechanism cannot sufficiently lower the intraorbital pressure, occlusion of the central retinal artery and/or vascular supply of the optic nerve can occur. After ~60 minutes, this results in irreversible ischemia of the retina and/or optic nerve, leading to blindness.^11^ Clinical signs of OCS include severe ocular pain with tense and swollen eyelids, progressive exophthalmos, acute vision loss, restricted eye movement, and a relative afferent pupillary defect (RAPD). Secondary to increased intraorbital pressure, IOP may also rise. The treatment of OCS consists of urgent orbital decompression through a lateral canthotomy and caudal cantholysis.^12^ In cases of more gradual intraorbital lesion growth, such as the encephalocele in the present case, compensatory exophthalmos is often sufficient to prevent OCS.

In conservatively managed orbital roof fractures, regular ophthalmologic examinations are warranted, including monitoring of IOP, periorbital swelling, exophthalmos, ocular alignment, and the presence of RAPD to detect (incipient) opticopathy. In conscious patients, visual acuity, color vision, and ocular motility should also be assessed. If new ophthalmologic abnormalities are observed, imaging should be repeated. A TIOE is a rare complication of an orbital roof fracture, in which brain parenchyma herniates through a dural defect at the fracture site.^13–19^ This typically presents with (pulsatile) exophthalmos. A review by Cammarata et al^15^ analyzed 28 cases of orbital roof fractures complicated by a TIOE. In 20 patients, TIOE developed within a month post-trauma. This is referred to as acute/early TIOE and develops due to intracranial swelling exerting downward pressure on the orbital roof. In the other 8 patients, TIOE was diagnosed more than a month post-trauma. The subacute/chronic TIOE likely shares pathophysiological similarities with growing skull fractures seen in pediatric patients. Over time, the continuously pulsating brain parenchyma gradually herniates through the orbital roof fracture.^20^ Only one patient included in the review was managed non-surgically. Similar to the present case, clinical and radiological resolution of the encephalocele was observed in the early phase as intracranial swelling subsided.^21^

Orbital roof fractures complicated by a TIOE require a multidisciplinary approach involving neurosurgery, oral and maxillofacial surgery, and ophthalmology. If surgical repair is warranted, access to the orbital roofs can be achieved through a bicoronal incision followed by a frontal craniotomy and careful retraction of the frontal lobes. The contused intraorbital parenchyma is then excised, and a dural repair (duraplasty) is performed to restore the watertight barrier between the intraorbital and intracranial compartments. Reconstruction of the orbital roof can be performed through a transcranial or transorbital approach. Transorbital reconstructions are typically performed using a titanium mesh, which can be preshaped on a 3D-printed mold. Transcranial reconstructions can be performed using autologous bone (calvarial bone, tabula interna) or alloplastic materials. Titanium is preferred due to its complex geometry, excellent biocompatibility, and resistance to resorption.^3^

## CONCLUSION

Orbital roof fractures are relatively uncommon facial fractures, typically seen after high-force trauma in combination with intracranial injury and other facial fractures. International consensus regarding the management of these fractures has yet to be achieved. Surgical reconstruction should be considered in cases of threatened visual function, severe displacement of fracture fragments, additional facial fractures requiring repositioning, and dural laceration. A rare complication of an orbital roof fracture is a TIOE, in which brain parenchyma herniates through the fracture site. This often presents as a (pulsatile) exophthalmos and may lead to compression of intraorbital structures and/or increased intraorbital pressure. The treatment strategy for a TIOE should be determined through a multidisciplinary approach. This article describes the case of a blow-in fracture of the orbital roof complicated by a TIOE in the early phase. As intracranial swelling subsided, spontaneous repositioning of the orbital roof and resolution of the TIOE were observed, followed by remodeling in good alignment. Because of its sporadic occurrence, little is known about the natural course of a TIOE. In clinically stable patients without signs of a threat to visual function, a conservative approach with close monitoring of ophthalmologic functions seems justified in the early phase while intracranial swelling subsides, pending potential spontaneous repositioning.
